# Identification of Genes Associated with the Pan-Vibrios Resistance (PVR) Trait of Pacific White Shrimp (*Litopenaeus vannamei*) Using a Genome-Wide Association Study

**DOI:** 10.3390/biology15030208

**Published:** 2026-01-23

**Authors:** Shuyang Wen, Chuhang Cheng, Jiayue Yin, Ying Lv, Xin Zhang, Bo Ma, Yang Liu, Yueshan Qiu, Huteng He, Peng Luo, Lihong Yuan

**Affiliations:** 1 School of Life Sciences and Biopharmaceutics, Guangdong Pharmaceutical University, Guangzhou 510006, China; 2 Key Laboratory of Breeding Biotechnology and Sustainable Aquaculture (KLBBSA), Guangdong Provincial Key Laboratory of Applied Marine Biology (LAMB), South China Sea Institute of Oceanology, Chinese Academy of Sciences, Guangzhou 510301, China; 3 Guangxi Key Laboratory of Marine Environmental Science, Guangxi Academy of Marine Sciences, Guangxi Academy of Sciences, Nanning 530007, China; 4 Guangxi Key Laboratory of Beibu Gulf Marine Biodiversity Conservation, Beibu Gulf Marine Ecological Environment Field Observation and Research Station of Guangxi, College of Marine Sciences, Beibu Gulf University, Qinzhou 535011, China; 5 School of Sciences, Xi’an Jiaotong-Liverpool University, Suzhou 215123, China

**Keywords:** GWAS, *Litopenaeus vannamei*, pan-vibrios resistance, SNPs, *LvHEATR1* gene

## Abstract

Vibriosis, caused by diverse *Vibrio* species, is among the most devastating bacterial diseases in shrimp aquaculture. Consequently, breeding shrimp for pan-vibrios resistance (PVR) presents a crucial strategy for sustainable shrimp farming. In this work, we performed a GWAS in *Litopenaeus vannamei* to identify genetic loci underlying resistance to pan-vibrios and validate the identified SNPs. A total of 300 shrimp from nine different regions were subjected to a comprehensive challenge. Selective genotyping of 300 resistant and susceptible individuals was conducted using a specific length amplified fragment sequencing (SLAF-seq) approach. A total of 18,184,608 high-quality SNPs were detected across the whole genome of *L. vannamei*. Screening identified 283 SNPs located within genes, 26 of which were associated with the PVR trait. These SNPs were subsequently validated in verification group of 80 shrimps, leading to the identification of two genotypes (GG at SNP20 and AA at SNP21) and one genotype combination (GG/AA at SNP20 and SNP21) that were significantly associated with the PVR trait. Notably, these linked SNPs were identified in the intron of *LvHEATR1* gene. The highest *LvHEATR1* expression was observed in immune-related tissues including hemocytes, the gills, and the hepatopancreas. Furthermore, qPCR results showed that *LvHEATR1* expression was significantly higher in the vibrios-resistant (RES) group than in the vibrios-susceptible (SUS) group. This study proposed the PVR concept and provided valuable molecular markers for the genetic improvement of vibrios-resistance in *L. vannamei*.

## Simple Summary

The Pacific white shrimp, *Litopenaeus vannamei*, is the most extensively cultivated shrimp species worldwide, accounting for over 75% of global production. However, intensive farming practices have frequently led to outbreaks of bacterial diseases, predominantly caused by various pathogenic *Vibrio* species, resulting in substantial economic losses. Current breeding efforts have largely focused on developing shrimp resistance to individual *Vibrio* species. Given the high diversity of *Vibrio* species, resistance to a single *Vibrio* species is insufficient to address the complex environments of aquaculture. Therefore, breeding shrimp with pan-vibrios resistance (PVR) has emerged as a crucial strategy for achieving sustainable shrimp aquaculture. This study aims to identify single nucleotide polymorphisms (SNPs) associated with PVR traits and pinpoint candidate functional genes using a genome-wide association study (GWAS), with subsequent validation. The results of the study are of great significance for promoting healthy aquaculture development and the sustainable development of marine fishery resources.

## 1. Introduction

Pacific white shrimp, *Litopenaeus vannamei*, is the most widely farmed shrimp species globally, accounting for over 75% of total production [1]. However, with the widespread farming of the shrimp, vibriosis caused by various pathogenic *Vibrio* species frequently break out, resulting in severe production and economic losses [2,3,4]. Current strategies to control vibriosis primarily rely on antibiotics [2,3], immunostimulants [4,5], and genetic breeding approaches [6,7,8]. Among these, genetic breeding is regarded as the most promising strategy, as it can fundamentally enhance shrimp resistance to *Vibrio* proliferation through genetic improvement [8].

In recent years, high-throughput sequencing technologies have significantly advanced the genetic breeding of aquatic animals. Molecular-marker-assisted selection (MAS), which mainly depends on mining molecular markers from high-throughput sequencing and data analysis, has been widely applied in several aquatic animals, such as *Crassostrea Gigas*, *Cyprinus carpio*, *Larimichthys Crocea,* and *L. vannamei* [9,10,11,12]. Genome-wide association study (GWAS) plays an important role in mapping complex traits and in the identification of genetic variants at the genome-wide scale [13]. GWAS has been used to identify important loci for MAS, aiming to enable genetic selection of multiple traits in animals [14]. In genetic breeding of shrimp, for instance, some single nucleotide polymorphisms (SNPs) associated with WSSV resistance [15], growth traits, and sex determination [16] have been identified in *Penaeus monodon* through GWAS. Medrano-Mendoza et al. [17] first analyzed the correlation between the WSSV resistance trait and SNPs in *L. vannamei* using GWAS. Liu et al. [10] identified seven genes related to ammonia nitrogen tolerance in *L. vannamei* using GWAS. Whankaew et al. [18] identified four SNPs and 17 InDels in three varieties of *L. vannamei* related to the tolerance to Acute Hepatopancreatic Necrosis Disease (AHPND). However, to date, no further screening and validation of candidate SNPs identified by the GWAS for the vibrios-resistance in shrimp has been conducted. In addition, nearly all SNPs associated with vibrios-resistance in shrimp were identified to target the genetic improvement of AHPND tolerance, wherein only specific virulent strains of *Vibrio* species (such as *V. parahaemolyticus*) were adopted [7,8,19]. *Vibrio* is a genus of ubiquitous bacteria found in a wide variety of aquatic and marine habitats, and at least 140 species have been recorded in this genus https://www.bacterio.net/genus/vibrio (accessed on 10 January 2026), among which more than 16 *Vibrio* species have been reported as opportunistic pathogens in shrimp [20]. Due to the prevalence of pathogenic *Vibrio* species in diverse marine environments, genetic breeding of shrimp with the pan-vibrios resistance (PVR) trait (i.e., resistance to wide range of *Vibrio* pathogens) has more practical significance for successful shrimp farming.

In the present study, a GWAS of the PVR trait was performed in 300 shrimp from different sources. SNPs associated with PVR were identified, providing insights into *L. vannamei* resistance to pan-vibrios. Genotypes of these candidate SNPs were differentiated in a validation group and their association with the PVR trait was analyzed. Finally, a genotype combination in *LvHEATR1* that was strongly associated with the PVR trait was identified. This study proposes the concept of the PVR trait and provides valuable molecular markers for genetic selection in *L. vannamei*, facilitating the breeding for improved vibrios-resistance.

## 2. Materials and Methods

### 2.1. Shrimp Sample Collection

In this study, 510 shrimps were divided into two populations: the experimental population and the verification population. The experimental population consisted of 300 shrimps (weight 11 ± 4 g), which were collected from 9 regions in China (Sanya, Qionghai, Fuzhou, Xiamen, Ningde, Zhanjiang, Zhuhai, Shantou, and Guangzhou). The verification population consisted of 240 shrimps collected from 4 farms in Maoming, China. The two populations were separately raised in two identical ponds in Maoming, China, with the ponds filled with 10 tons of natural seawater (25 parts per thousand, pH 8.1), and the seawater temperature was maintained at 27 °C ± 2 °C, under a 12 h dark–12 h light photoperiod. The shrimps were fed twice a day, in the morning and afternoon, with commercial feed (HengXing, Zhanjiang, China). The load of vibrios in the water was confirmed by a TCBS test to be 10^3^ CFU/L, and this setup ensured sufficient contact between the shrimps and the widely existing bacteria. One week after the cultivation, samples of the shrimps were collected for experiments. The surface of the shrimp was rinsed with deionized water and wiped with 75% alcohol to prevent bacterial infection. Then, the carapace was removed, and the liver and pancreas surface was lifted using sterilized forceps to collect the internal tissues of the shrimps. A total of 0.1 g of these tissues was placed in an EP tube for TCBS testing, while the remaining parts were quickly frozen in liquid nitrogen for subsequent DNA extraction.

### 2.2. Evaluation of the PVR Trait by the Load of Vibrios in Hepatopancreas

To classify the PVR levels in shrimp, the load of vibrios in the hepatopancreas was quantified using Thiosulfate Citrate Bile Salts Sucrose (TCBS) agar, and the resistance levels were categorized based on the load of vibrios. A total of 0.1 g of hepatopancreas tissue was placed into the EP tube and homogenized with 1 mL of sterile seawater. The hepatopancreas homogenate was then subjected to a 10-fold serial dilution. A total of 100 μL of hepatopancreas homogenate from the 1×, 10×, and 100× dilutions were spread evenly onto TCBS plates. The plates were incubated at 30 °C for 24 h, after which the colony-forming units (CFUs) were enumerated. Shrimp individuals were classified into five grades (G1, G2, G3, G4, and G5) according to the load of vibrios to reflect their PVR trait, among which the severity of *Vibrio* proliferation progressively escalates from grade 1 to grade 5 (G1 to G5). Finally, for the validation group, a total of 80 resistant individuals (G1) and susceptible individuals (G4 and G5) were selected from the 240 shrimp based on their hepatopancreas the load of vibrios levels.

### 2.3. DNA Extraction and Library Preparation

The genomic DNA of each shrimp sample was extracted using the Marine Animals Genomic DNA Extraction Kit (TIANGEN, Beijing, China). All 300 shrimp from nine regions in the experimental population were used for GWAS detection. SLAF-seq sequencing was conducted, strictly according to the methods described in reference [21]. The sequencing platform adopted was Illunima Hiseq 2500 (Illunima, San Diego, CA, USA), and the average sequencing depth was 11.04×.

### 2.4. Genotyping and Quality Control

Filtered reads were mapped to the reference genome of *L. vannamei* (ASM 378908) using BWA software v0.7.19 [22]. GATK v4.2 [23] and SAMtools v1.19.2 [24] were used to develop the SNP tag intersection as the final reliable SNP tag dataset. Subsequently, SnpEff software v5.0 [25] was used to analyze the mutation sites and gene information in the reference genome. The “*check marker*” function in the GenABEL package was used for quality control of genotyping SNPs in an R environment [26]. The SNPs were identified as having a minor allele frequencies (MAF) > 0.05, Max-missing < 0.2, or Hardy-Weinberg equilibrium < 1 × 10^−6^. In addition, SNPs located within genes were focused on. Then, genome-wide significance was assessed by the Bonferroni method, in which the conventional *p* value was divided by the number of tests performed (SNPs tested).

### 2.5. Variant Calling Analysis and GWAS

Combined with EMMAX [27], the following mixed linear model was used for correlation analysis:

y=Wα+xβ+μ+e

where y represents phenotypic data, W is the population structure variable, x is the genotype data, α and β are the effect values corresponding to W and x, and e is the residual. According to the *p*-value of the SNP, with −log10 (*p*) ≥ 2 as the significance standard, the corresponding Q–Q quantile map and Manhattan map were drawn by using the R qqman package v0.1.9 (https://doi.org/10.32614/CRAN.package.qqman).

### 2.6. Function Analysis of Genes Containing Discrepant SNPs and Genotyping of Discrepant SNPs in the Validation Group

Genes containing discrepant SNPs identified by the GWAS were screened based on SNP locations using the genomic information of *L. vannamei* (ASM 378908v1; the genome used is not the latest reference genome), and gene functions were annotated using the NCBI-NR database. The tag sequence information for the selected genes was obtained from the *L. vannamei* genome. Primer premier 6.0 software (Applied Biosystems, Norcross, GA, USA) was used to design primers for PCR, targeting DNA fragments near the candidate SNP. Two forward primers were designed for each site, and fluorescent sequence tags FAM (GAAGGTGACCAAGTTCATGCT) and HEX (GAAGGTCGGAGTCAACGGATT) were added. All primers used in this study were designed by Primer premier 6.0 software and are listed in Table S1. Genotyping was performed using competitive allele specific PCR (KASP) with a commercial STO Rox kit (Gudebio, Guangzhou, China).

### 2.7. Correlation Analysis Between Genotypes and the Performance of the PVR Trait in the Validation Group

The load of vibrios of 240 shrimp from the Maoming farm was detected using TCBS plates. Among them, a total of 80 shrimp with the load of vibrios belonging to G1 were classified as the vibrios-resistant (RES) group, while G4 and G5 were classified as the vibrios-susceptible (SUS) group. These shrimps will be used to validate the candidate SNPs. The association between SNPs and trait was identified by SPSS 27 software https://www.ibm.com/cn-zh/products/spss (accessed on 15 August 2025). Among them, SNP loci with *p* < 0.05 were considered to be significantly associated with the trait of PVR and the association between the genotypes of these SNPs and the load of vibrios was examined. SNPs significantly associated with the PVR trait were further used to explore potential interactions among them using linkage disequilibrium (LD) analysis and Haploview software v4.2 [28]. Based on the LD analysis, the correlation between the genotype combination and the PVR trait performance was also analyzed, leading to the identification of combined SNP markers.

### 2.8. Protein Structure Prediction of a Target Gene Related to the PVR Trait and Expression Levels of the Gene

The SWISS-MODEL website https://swissmodel.expasy.org/ (accessed on 26 August 2025) was used to predict the 3D structure of the protein coded by a target gene related to the PVR trait. Total RNA was extracted using an RNA Easy Fast Tissue/Cell Kit (TIANGEN, Beijing, China) and was reverse-transcribed into cDNA using a Prime Script™II 1st Strand cDNA Synthesis Kit (Takara, Tokyo, Japan). The expression levels of the gene in the brain, eye stalk, hemocytes, gill, hepatopancreas, heart, muscle, intestine and stomach were detected by qRT-PCR using a RT-PCR kit (TaKaRa, Tokyo, Japan) and the *β-actin* gene was used as the reference gene (Table S1). qRT-PCR was performed using Thermal Cycler Dice^®^Real Time System III (TaKaRa, Tokyo, Japan) under the following conditions: 95 °C for 30 s, 40 cycles of 95 °C for 5 s and 56 °C for 30 s, reading of templates at 95 °C for 15 s, and a final melting curve from 60 to 95 °C. Three biological replicates and three technical replicates were used for qRT-PCR, and gene expression levels were calculated by the 2^−ΔΔCT^ method [29].

### 2.9. Prediction of Splicing Sites

NetGene2 v2.42 https://services.healthtech.dtu.dk/ (accessed on 28 July 2025) was used to predict SNP loci in intronic sequences and splicing sites (confidence ≥ 0.80).

## 3. Results

### 3.1. Grading the PVR Trait of the Shrimp Individuals

**Figure 1:**
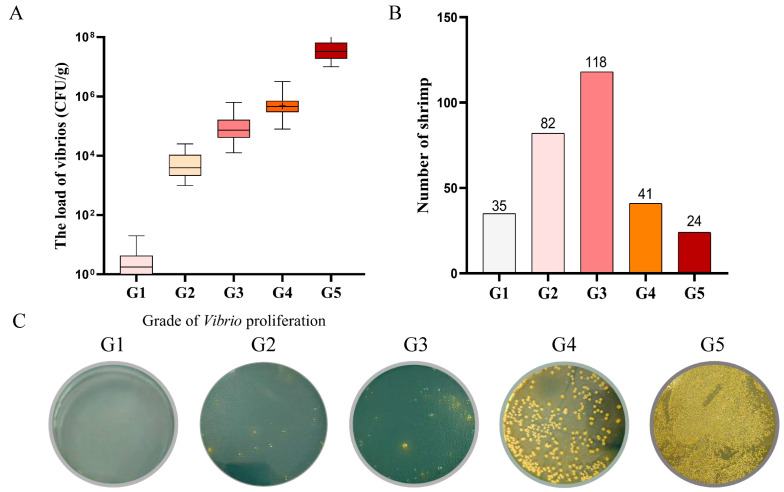
The load of vibrios corresponding to different proliferation grades. (**A**) The load of vibrios corresponding to different proliferation grades in the test group. (**B**) Number of individuals in each proliferation grade. (**C**) Representative bacterial colonies on TCBS plates for each proliferation grade.

According to the load of vibrios in the hepatopancreas, the PVR trait of the shrimp was quantified to five grades, G1 to G5 (Figure 1A). Among 300 shrimp, only 25 individuals in G1 (8.3%) exhibited significant PVR traits, while 32 individuals in G5 (10.7%) exhibited extensive proliferation in hepatopancreas, weakly indicating the PVR trait performance (Figure 1B). The typical bacterial colonies of different proliferation grades on the TCBS plates are shown in Figure 1C.

### 3.2. GWAS and Gene Functional Annotation of the Candidate SNPs

**Figure 2:**
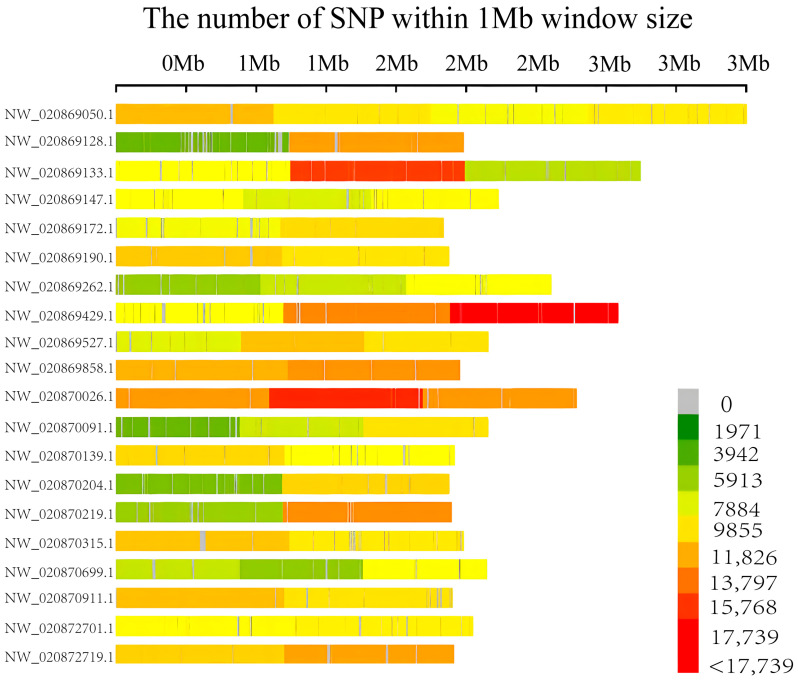
Distribution of SNPs across the chromosomes on the 20 longest scaffolds in *L. vannamei*. The color bar indicates the number of SNPs within 0.1 Mb window, with the index shown on the right.

According to the comparison analysis, 18,184,608 SNP loci were identified, 78.78% were located in intergenic regions, whereas only 5.95% were located within genes. Moreover, these SNPs were unevenly distributed across the genome, with the highest densities on scaffolds, NW_020869133.1, NW_020869429.1, and NW_020870026.1 (Figure 2).

**Figure 3:**
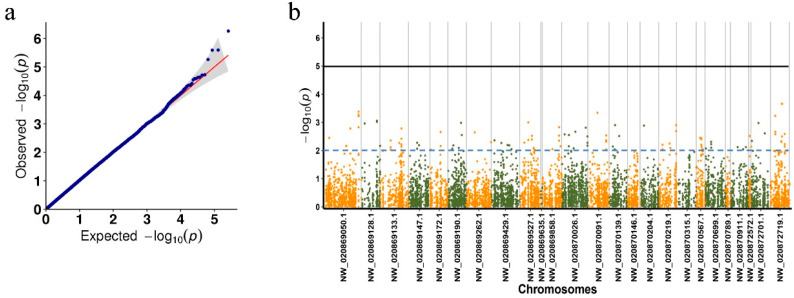
GWAS for the shrimp PVR trait. (**a**) Q–Q plot and (**b**) Manhattan plot. The dashed blue line and solid black line indicate −log10(*p*) = 2 and −log10(*p*) = 5, respectively. Since the genome of *L. vannamei* is not assembled at the chromosomal level, in this study, only SNP distributions on the 20 longest scaffolds are visualized.

The Q–Q plot indicated that the test statistics were well controlled after effective correction for family structure, indicating the phenotypic data fit the selected model well (Figure 3a). The Manhattan plot showed the GWAS results, with a total of 1939 SNPs above the dotted significance threshold (−log10 (*p*) ≥ 2, Figure 3b). After focusing on SNPs inside genes (distance < 1), a total of 283 SNPs potentially related to the PVR trait were identified. These genes encompassed a variety of gene functions, including immune regulation, metabolism, cell cycle, and signal transduction (Table S2). We selected 10 immune-related genes, including a total of 26 SNPs for validation (Table 1). These genes included Toll pathway signal transducers, immune recognition receptors, caspases, protease inhibitors, and chitinases.

### 3.3. Genotyping of the SNPs and Validation of the Candidate SNPs and Genotypes in the Validation Group

The load of vibrios within the 240 shrimp collected from Maoming was determined using TCBS agar plates. Based on the results, RES (G1, *n* = 26) and SUS (G4 and G5, *n* = 54) individuals were selected to form a validation group for genotyping 26 candidate immune-related SNPs. The genotypes of these SNPs were statistically analyzed to identify those exhibiting significant differences between the RES and SUS groups. A total of five SNPs (SNP1, SNP6, SNP20, SNP21, SNP22) showed significant differences between the two groups (*p* < 0.05). Although the genotype profiles of SNP1 and SNP6 showed a significant difference between the two groups, no valuable genotypes were found to be associated with the PVR trait. The GG genotype of SNP20, the AA genotype of SNP21, and the TT genotype of SNP22 were more frequent in the RES group (Table 2). However, the AA genotype of SNP1 (64.0% in the SUS group) and the AA genotype of SNP6 (85.7% in the SUS group) showed a significant correlation with pan-vibrios susceptible (PVS) traits. Consequently, SNP1 and SNP6 cannot be regarded as reliable molecular markers for the PVR trait and were more likely associated with the PVS trait.

**Figure 4:**
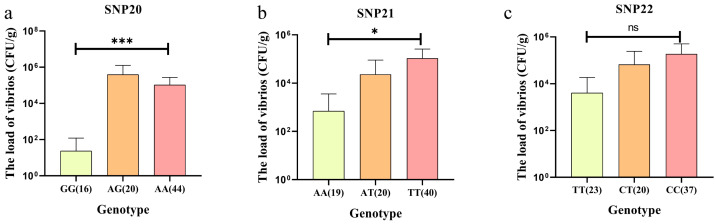
The correlation between genotypes of the three SNPs and the load of vibrios in the validation group. (**a**) the association between SNP20 genotype and the load of vibrios. (**b**) the association between SNP21 genotype and the load of vibrios. (**c**) association between SNP22 genotype and the load of vibrios. Data were analyzed using ANOVA, where * indicates *p* < 0.05 and *** indicates *p* < 0.001. “ns” indicates no significant difference between groups. The numbers in parentheses after the genotypes represent the number of individuals with that genotype.

As a result, these three SNPs were selected as candidate loci for subsequent validation. Statistical analysis of the correlations between the genotype of the three SNPs and the load of vibrios showed that only the genotype of SNP20 and SNP21 had significant differences in the load of vibrios. The GG genotype of SNP20 (<10^2^ CFU/g) and AA genotype of SNP21 (<10^3^ CFU/g) had the lowest load of vibrios and were significantly different from other genotypes (*p* < 0.05, Figure 4a,b), indicating that the GG genotype of SNP20 and the AA genotype of SNP21 are significantly associated with the PVR trait. Although no statistically significant association was observed between SNP22 genotypes and the load of vibrios, individuals carrying the TT genotype of SNP22 exhibited lower the load of vibrios compared to other genotypes (Figure 4c).

### 3.4. Haplotypes of the SNPs and Correlation Analysis Between the Haplotypes and PVR Trait

**Figure 5:**
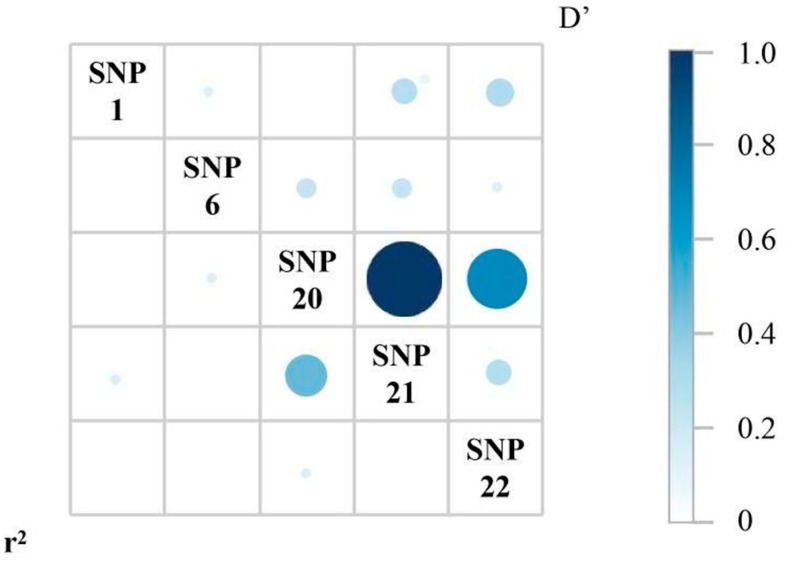
Linkage disequilibrium (LD) analysis of the five SNPs. Pairwise LD is shown as a matrix. The circle in the lower left matrix represents the r^2^ value, and the circle in the upper right matrix indicates the D’ value (as shown by the color scale on the right).

Linkage disequilibrium (LD) analysis was performed on the five SNPs. The results showed strong linkage disequilibrium between SNP20 and SNP21 (Figure 5), and significant differences in the GA and AT haplotypes of SNP20 and SNP21 between the RES and SUS groups. Specifically, the GA haplotype was more prevalent in the RES group (72.7%), whereas the AT haplotype was more common in the SUS group (86.6%, Table 3).

**Figure 6:**
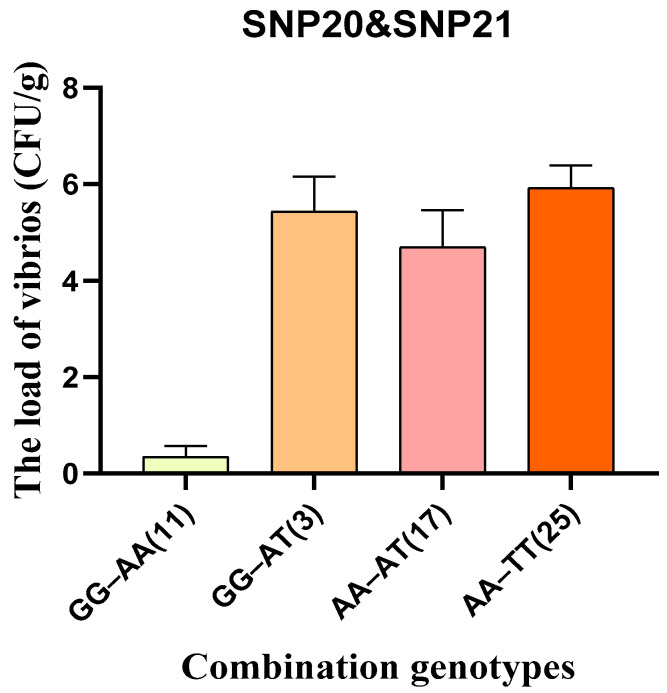
The correlation between combination genotypes of SNP20 and SNP21 and the load of vibrios in the validation group. The numbers in brackets indicate the number of individuals with the combined genotype.

Statistical analysis of the load of vibrios showed that individuals with the GA haplotype had a significantly lower the load of vibrios than those with the AT haplotype. In addition, to enhance the development of molecular markers, GA haplotypes and AT haplotypes were further classified into four genotype combinations, GG/AA, GG/AT, AA/TT and AA/AT (Figure 6). Among these, individuals with the GG/AA genotype combination (derived from the GA haplotype) exhibited the lowest the load of vibrios, while those with the AA/TT combination (derived from the AT haplotype) showed the highest. Using homozygous genotypes may help reduce trait segregation during breeding.

### 3.5. Structure Prediction and Tissue Distribution of the Gene Harboring the Three SNPs

**Figure 7:**
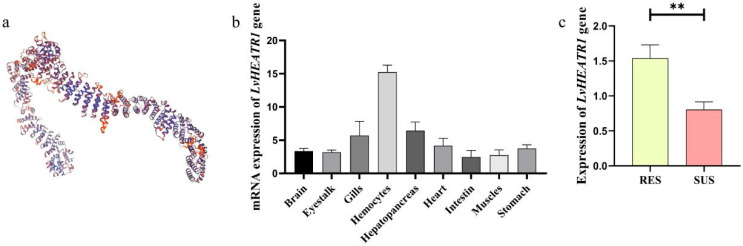
Analysis of the *LvHEATR1* gene function. (**a**) the 3D structure of protein encoded by the gene *LvHEATR1* (**b**) the expression distribution of *LvHEATR1.* (**c**) mRNA expression of *LvHEATR1* gene in the RES and the SUS group. “**” indicates that this gene shows a significant difference between the RES group and the SUS group (*p* < 0.01).

The three SNPs (SNP20, SNP21, SNP22) were located in intronic region of *LvHEATR1*, which encodes for a collagen triple helix repeat containing protein 1. The 3D protein structure prediction showed that it contains many repeated alpha helices, accounting for about 70.64% of the gene (Figure 7a), which may be closely related to its function. The expression level of *LvHEATR1* was relatively high in hemocytes, the hepatopancreas, and the gills (Figure 7b), and expression was highest in hemocytes (approximately fivefold higher than in muscle). Moreover, *LvHEATR1* expression was significantly higher in the RES group than in the SUS group, suggesting a potential association between *LvHEATR1* and the PVR trait in *L. vannamei* (Figure 7c).

### 3.6. Variations in SNPs Changed the Position of Predicted Splice Sites

**Figure 8:**
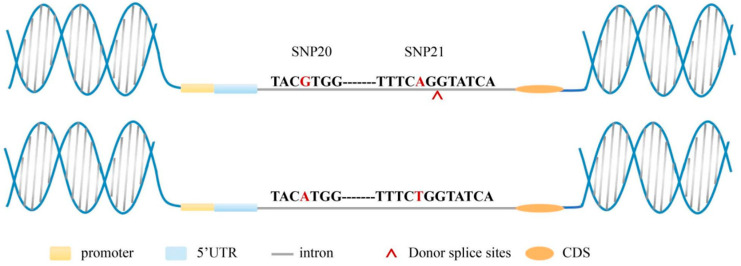
Prediction of the altered splicing site in the genotype combination. The bases with red color are SNP20 and SNP21. The gene sequence above represents the GG/AA combined genotype, and the gene sequence below represents the AA/TT combined genotype. Donor splicing sites that were identified in the region between SNP20 and SNP21 of GG/AA.

A total of six splice sites were identified, comprising four splicing donor sites and two splicing acceptor sites. Notably, the GG/AA genotype combination of SNP20 and SNP21 contained one additional recognizable donor splice site compared to the AA/TT genotype combination (Table 4), which is predicted to generate a novel transcript and consequently affect protein structure and function. This splicing donor site is located behind SNP21 and close to SNP21 (Figure 8).

## 4. Discussion

*Vibrio*, as a common aquatic bacteria, have caused serious harm and great economic losses in shrimp culture [30,31]. There is a high diversity and ubiquity of *Vibrio* species in estuarine and marine environments [32,33], and *Vibrio* is classified as opportunistic and conditionally pathogenic bacteria; for instance, an increase in temperature can damage the metabolic pathways and adaptability of *Vibrio harveyi*, where the expression of virulence genes is upregulated [34]. Therefore, shrimps with a higher colonization rate are more likely to be infected by vibrios and suffer from related diseases. Given these conditions, the concept of PVR was proposed; these shrimps can resist a wide range of *Vibrio*, including both pathogenic and non-pathogenic *Vibrio*, in the aquaculture environment. The objective is to identify and select shrimp capable of resisting a broad spectrum of vibrios proliferation, thereby reducing the risk of vibrios diseases in shrimp aquaculture. Likewise, it is likely that breeding materials of shrimp screened through the infection challenge by single or multiple *Vibrio* species may not be capable of adapting to the complicated farming environments full of various vibrios [35,36]. Based on this consideration, we were inclined to obtain ideal breeding materials for shrimp from actual farming environments instead of the infection challenge by specific *Vibrio* species.

In this study, the shrimp were continuously exposed to a diverse range of vibrios conditions throughout their growth period, which indicates that they have taken up the challenge of mixed strains of vibrios. By farming the shrimp in seawater containing *Vibrio*, it is ensured that all individuals are adequately exposed to the *Vibrio*, including pathogenic and non-pathogenic *Vibrio*, thereby avoiding false-resistant shrimp. The hepatopancreas of shrimp is not only a digestive organ but also an immune organ of shrimp that is frequently invaded by pathogenic bacteria, viruses, and parasites. Diversified vibrios can easily be found in the farmed shrimp, *L. vannamei.* In long practices of farming and pathological examinations, we found that the load of vibrios in the hepatopancreas can reflect the healthy state and resistance ability of the shrimp, and similar findings have also been reported by other researchers [36,37]. For another parasite pathogen, *Enterocytozoon hepatopenaei* (EHP), EHP load in the hepatopancreas of *L. vannamei* also reflects the resistance ability of the shrimp [38,39,40]. In this study, we used TCBS plating to calculate the load of vibrios in hepatopancreas of shrimps. It is well known that some other bacteria such as *Photobacterium* and *Pseudoalteromonas* species can also grow on the TCBS plates [41,42]. However, TCBS plating are still used as the conventional method to calculate the number of vibrios in laboratory and farming practices [43,44,45] as this method is very convenient and vibrios can form dominant colonies within overnight culture (less than 24 h). In addition, viable-but-non-culturable (VBNC) state is another issue that may affect the calculation of vibrios. VBNC state is a self-protection mechanism of some bacteria such as vibrios [46,47]. Under natural conditions, bacteria enter VBNC due to a decrease in temperature or a lack of nutritional environment [48,49]. However, during shrimp farming and TCBS cultivation, we ensured that vibrios had suitable temperatures and sufficient nutrients and all the hepatopancreas samples were treated under the same conditions. Therefore, even though some vibrios may have entered a VBNC state, it does not affect the final evaluation of the PVR trait measured by the load of vibrios. Based on these reasons, in this study, the load of vibrios in the hepatopancreas of *L. vannamei* can be determined by TCBS plating, thereby quantifying the PVR trait, and the subsequent screening of the final resistance SNP markers can be linked to the performance of the PVR trait.

The shrimp individuals with strong PVR traits were approximately 10% in the test group and the validation group, which indicates that only a few individuals in a shrimp group have strong resistance to *Vibrio* proliferation, likely conferred by the genetic variations in these individuals. This explains the relatively small number of G1 individuals in the shrimp population used in the experiment, and this result is consistent with natural principles.

GWAS is a powerful tool for association analysis between genotypes and phenotypes without breeding information. It plays a significant role in the screening of resistance to vibrios in aquatic animals. Currently, 18 SNP loci associated with resistance to vibrios in oysters have been identified [50], and seven SNPs linked to resistance against *Vibrio harveyi* in yellow croaker have also been reported [51,52]. Additionally, four SNPs related to vibrios-resistance have been identified in shrimp. However, at present, this resistance screening is restricted to specific pathogenic *Vibrio* species and has not been further validated for SNPs. In this study, whole genomes of 300 shrimp containing five different PVR levels (G1 to G5) were sequenced for GWAS. Through GWAS, 26 SNPs were first screened out, and these SNPs were annotated to the genes related to Toll pathway signal transducers, immune recognition receptors, caspases, protease inhibitors, and chitinases. After KASP genotyping, three SNPs (SNP20, SNP21, SNP22) were further screened out to be likely associated with the PVR trait and two SNPs (SNP1, SNP6) were associated with PVS traits and do not have breeding value. Validation of the association between genotype and the load of vibrios confirmed that the GG genotype of SNP20 and the AA genotype of SNP21 are significantly associated with the PVR trait, thereby providing support for molecular marker breeding.

In addition, LD analysis is an important method to examine SNP–SNP interaction, which has been shown to play an important role in the selection of species for complex traits [40,41,42]. Furthermore, both SNPs located in the intron region of the *LvHEATR1* gene also supported the LD of the two SNPs, which propelled the potential genotype combination that is closely associated with the PVR trait. The application of combined molecular markers narrows the screening range and increases the accuracy of breeding materials [28], which also theoretically matches the rule that most economic traits are controlled by a set of loci in a genome [53,54]. *LvHEATR1* is one kind of HEAT repeat protein, which includes a mammalian target of rapamycin (mTOR) protein, owning a common function of mediating protein–protein interactions [55]. mTOR plays an important role in various cellular activities, such as immunity, and it is activated by the formation of polymers in mammalian cells through its N-terminal HEAT repeat region [56]. In addition, a HEAT repeat protein, *ILITYHIA* (*ILA*) in *Arabidopsis,* involved in the plant immune process, and the mutation of *ILA*, leads to increased sensitivity to pathogens and systemic drug resistance defects in plants [57]. In addition, the mTOR proteins of crustaceans, such as *Gecarcinus lateralis* and *Carcinus maenas*, also contain the HEAT repeat structure [58]. The genes involved in the mTOR pathway of *L. vannamei* are strongly associated with the bacterial resistance phenotype exhibited by these crabs [59,60]. The expression distribution of *LvHEATR1* in the shrimp showed that the mRNA was highly expressed in immune-related tissues such as hemocytes, the gill, and the hepatopancreas, which are also frequently attacked by vibrios. The RES group exhibited significantly higher expression levels of the *LvHEATR1* gene compared to the SUS group, suggesting a potential association between this gene and the PVR trait in shrimp. Therefore, it can be speculated that *LvHEATR1* may play a crucial role in the immune process of *L. vannamei*, although the specific function of HEAT repeat proteins in shrimp has not been elucidated.

Generally, introns can’t be transcribed into mRNA, and they are often considered as junk DNA regions [61]. However, recent studies have shown that introns are closely related to gene expression and cytoskeleton construction and exert some influence on life activities [62,63]. InDels within introns in different cattle groups are extremely enriched in immune-related pathways [64]. Changes in body weight due to the SNPs in an intron of the *RuvBL2* gene have also been found in shrimp [65]. SNPs in introns can also lead to the occurrence of different spliceosomes sourced from the same gene [62,66,67]. In this study, the SNP loci result in alterations to the splicing donor site. Compared to the GG/AA combined genotype, the AA/TT genotype lacks a donor splice site. Based on this established correlation between the combination genotype and splice site alteration, we hypothesize that the loss of this splice site may lead to the production of a novel, nonsensical transcript during RNA processing. As a result, this novel transcript fails to produce a functional protein, ultimately contributing to the stronger PVR trait observed in GG/AA individuals compared to individuals with AA combination genotype. It is also possible that the donor splice site is important for shielding unspliced transcripts from degradation [68]. Consequently, its absence may lead to nonsense-mediated decay, thereby reducing *LvHEATR1* gene expression levels in the SUS group compared to the RES group. However, the precise splicing pattern and sequence of this putative novel transcript remain unclear. The existence of this transcript and its functional impact require further experimental validation.

## 5. Conclusions

In this study, the correlation between SNPs and the PVR trait of *L. vannamei* was analyzed. A total of 26 SNPs potentially associated with the PVR trait were initially identified using GWAS. Association analysis between genotype and the load of vibrios revealed a significant link between the PVR trait and the GG genotype of SNP20 and AA genotype of SNP21. The genotype combination of GG/AA of the SNP20 and SNP21 was significantly associated with the strongest performance of the PVR trait, and these SNPs were found to be located in the intron region of a gene, *LvHEATR1*. This study provides valuable molecular markers for the genetic selection of the PVR trait in shrimp, *L. vannamei*.

## Figures and Tables

**Table 1:** Statistics of the SNPs associated with the PVR trait.

SNP	Position	*p* Value	−log10*p*	Allele	MAF	Gene ID	Annotation
SNP1	438,087	2.97 × 10^−4^	3.53	A/G	0.42	LOC113825230	Adapter protein that plays a role in different signaling pathways including TLRs and IL-1 pathways or innate antiviral induction signaling.
SNP2	438,107	2.97 × 10^−4^	3.53	A/G	0.42		
SNP3	438,042	5.75 × 10^−4^	3.24	A/G	0.42		
SNP4	444,669	1.78 × 10^−3^	2.75	T/C	0.41		
SNP5	438,048	2.45 × 10^−3^	2.61	G/T	0.29		
SNP6	437,912	3.83 × 10^−3^	2.42	G/A	0.3		
SNP7	437,864	4.63 × 10^−3^	2.33	C/T	0.36		
SNP8	438,072	5.12 × 10^−3^	2.29	C/G	0.29		
SNP9	295,147	4.11 × 10^−3^	2.39	T/C	0.49	LOC113829309	The secreted form comprising the ectodomain can bind to bacteria and may act as an opsonin, enhancing their phagocytosis by hemocytes.
SNP10	364,053	9.81 × 10^−3^	2.01	A/T	0.48		
SNP11	364,054	9.81 × 10^−3^	2.01	T/G	0.48		
SNP12	21,376	9.38 × 10^−3^	2.03	C/T	0.48	LOC113805778	Involved in the activation cascade of caspases responsible for the execution of apoptosis.
SNP13	21,390	9.38 × 10^−3^	2.03	G/A	0.48		
SNP14	128,280	2.42 × 10^−3^	2.62	A/C	0.46	LOC113800227	Myeloperoxidase
SNP15	254,802	6.20 × 10^−3^	2.21	C/A	0.25	LOC113803300	Kazal-type protease inhibitor
SNP16	121,779	3.80 × 10^−3^	2.42	G/A	0.35	LOC113824913	Probable chitinase 2
SNP17	38,868	9.04 × 10^−3^	2.04	A/G	0.26	LOC113810647	Probable chitinase 10 isoform X1
SNP18	791,821	4.42 × 10^−3^	2.35	A/T	0.28	LOC113803006	Tachykinin-like peptides receptor 86C
SNP19	635,323	6.51 × 10^−4^	3.19	T/C	0.28	LOC113811875	HEAT repeat-containing protein 1-like
SNP20	635,082	1.41 × 10^−3^	2.85	G/A	0.45		
SNP21	635,340	2.55 × 10^−3^	2.59	T/A	0.41		
SNP22	635,341	2.65 × 10^−3^	2.58	G/A	0.41		
SNP23	635,106	3.87 × 10^−3^	2.41	T/C	0.41		
SNP24	162,844	9.42 × 10^−5^	4.03	C/T	0.47	LOC113817988	CUGBP Elav-like family member 2
SNP25	162,876	1.35 × 10^−3^	2.87	C/T	0.29		
SNP26	453,541	1.44 × 10^−3^	2.84	G/A	0.25	LOC113823610	Calpain-9-like

**Table 2:** Statistical analysis of SNPs associated with the PVR trait in the verification group.

SNP	Genotype	Number of Group		χ^2^	*p* Value
		RES (*n* = 26)	SUS (*n* = 54)		
SNP1	AA	12	31	6.390	0.041
	AG	5	19		
	GG	9	4		
SNP2	AA	2	0	4.54	0.110
	AG	23	50		
	GG	1	4		
SNP3	AA	6	4	4.31	0.130
	AG	17	44		
	GG	3	4		
SNP4	AA	0	0	0.58	0.392
	AT	2	2		
	TT	24	52		
SNP5	GG	1	3	0.68	0.443
	GT	4	12		
	TT	21	39		
SNP6	AA	6	27	8.20	0.017
	AG	14	24		
	GG	6	3		
SNP7	CC	10	22	2.97	0.246
	CT	10	27		
	TT	6	5		
SNP8	CC	22	43	1.64	0.424
	CG	2	9		
	GG	2	2		
SNP9	CC	20	45	0.929	0.379
	CT	3	6		
	TT	3	3		
SNP10	AA	14	34	0.469	0.791
	AT	7	12		
	TT	5	8		
SNP11	GG	17	45	0.219	0.898
	GT	6	13		
	TT	3	6		
SNP12	CC	5	9	0.106	0.948
	CT	15	33		
	TT	6	12		
SNP13	AA	19	48	1.18	0.602
	AG	0	3		
	GG	1	3		
SNP14	AA	25	46	2.37	0.126
	AG	1	5		
	GG	0	3		
SNP15	AA	15	35	8.147	0.939
	AG	4	4		
	GG	7	15		
SNP16	AA	0	0	ND	ND
	AG	26	54		
	GG	0	0		
SNP17	AA	18	31	1.44	0.478
	AG	6	18		
	GG	2	7		
SNP18	AA	18	36	0.74	0.963
	AT	6	13		
	TT	2	5		
SNP19	CC	0	6	2.42	0.139
	CG	0	0		
	GG	20	48		
SNP20	AA	10	34	16.51	2.6 × 10^−4^
	AG	4	16		
	GG	12	4		
SNP21	AA	13	6	15.58	4.1 × 10^−4^
	AT	2	17		
	TT	11	29		
SNP22	CC	9	28	12.35	0.020
	CT	3	17		
	TT	14	9		
SNP23	CC	12	34	2.11	0.151
	CT	13	19		
	TT	1	1		
SNP24	CC	19	45	2.04	0.185
	CT	5	8		
	TT	2	1		
SNP25	CC	6	4	4.26	0.135
	CT	2	3		
	TT	18	47		
SNP26	AA	6	17	1.84	0.414
	AG	15	32		
	GG	5	5		

“ND” indicates that there is no chi-square test result for this SNP.

**Table 3:** The load of vibrios associated with different combination genotypes in the validation group (*n* = 80).

LD	Haplotypes	Sample Size		χ^2^	*p*
		RES	SUS		
SNP20 and SNP21	AT	7	35	10.71	0.0011
	GA	11	3	11.33	8.0 × 10^−4^
	AA	6	12	0.001	0.974
	GT	2	4	2.01	0.156

**Table 4:** Prediction of splicing sites in the intron region of the *LvHEATR1* gene.

	Pos.5′-3′	Strand	Confidence	Recognition Sequence	
				**5′Exon**	**Intron3′**
Donor splicing sites	348	+	0.89	CCCATACGTG^GTATGACATT	
	453	+	0.88	CTTTGTCTTG^GTTTGTATTA	
	605 *	+	0.88	ATTGTTTCAG^GTATCACTTC	
	488	−	0.81	GTGGAGTTTG^GTAATTACCT	
				**5′Intron**	**Exon3′**
Acceptor splicing sites	316	−	0.81	CAATTTGCAG^AAGGGAAGTG	
	780	−	0.80	TGTTATTGAG^AGTGATGCAC	

* Bold represents a disappeared donor splicing site in the shrimp harboring the genotype combination of GG/AA. “^” represents the position for inserting the donor splicing site sequence.

## Data Availability

The data that support the findings of this study are available on request from the corresponding author. The data are not publicly available due to privacy or ethical restrictions.

## Supplementary Materials

The following supporting information can be downloaded at: https://www.mdpi.com/article/10.3390/biology15030208/s1, Table S1: Primers used in this study (DOCX) and Table S2: Candidate SNPs located within genes related to the traits of PVR (XLSX).

## Abbreviations

The following abbreviations are used in this manuscript:

GWAS	genome-wide association study
SNP	single nucleotide polymorphism
PVR	pan-vibrios resistance
PVS	pan-vibrios susceptible
LD	linkage disequilibrium
G1 to G5	grade 1 to grade 5
