# Supplementary material for: Identification of Genes Associated with the Pan-Vibrios Resistance (PVR) Trait of Pacific White Shrimp (Litopenaeus vannamei) Using a Genome-Wide Association Study
Source: Biology (Basel). 2026 Jan 23;15(3):208. doi: 10.3390/biology15030208 (PMC12896980; doi:10.3390/biology15030208)
Supplement: Supplementary file 1 [file biology-15-00208-s001.zip › Table S1.pdf]

Table S1 Primers used in this study

Primer	Position	Primer sequence 5'→3'
	438087F1	AGAGTAAACTCATTCGCCGAGCGGA
KASP1	438087F2	AGAGTAAACTCATTCGCCGAGCGGG
	438087R	ACACTCGGGCATGACCCCTCCTCGCCTTC
	438107F1	CATAAGACCATAAAAGAGCATTATT
KASP2	438107F2	CATAAGACCATAAAAGAGCATTATC
	438107R	TTTGGGGAGTCCGGGGTGGCCTCGCTCAC
	438042F1	GGTGGTTGCTCTCTTGGAGGTGACA
KASP3	438042F2	GGTGGTTGCTCTCTTGGAGGTGACG
	438042R	TAGCCACCTACACTAATCGTGCCACTGCC
	444669F1	CAACATCCCCGGCAAGATAAGGGCT
KASP4	444669F2	CAACATCCCCGGCAAGATAAGGGCC
	444669R	ATCGGGACTTTCCACACTCTCGAAGACGC
	438048F1	TTATCAGGAGAACTCTGCAGCCTTT
KASP5	438048F2	TTATCAGGAGAACTCTGCAGCCTTG
	438048R	AGTCTAAACTCTATCTCGCCTACAACATT
	437912F1	AAGTTTTTCTCTGGACTAATTTTAA
KASP6	437912F2	AAGTTTTTCTCTGGACTAATTTTAG
	437912R	TAACCGAAAAAATGACACATAGAAAACCT
	437864F1	AGAGGGAGAGAAAGAGTGAGAGAAT
KASP7	437864F2	AGAGGGAGAGAAAGAGTGAGAGAAC
	437864R	CTCGCTTTGTGTGTGTGTGTGTGTCTCTC
	438072F1	TAGTAAAAATGGGGTTTTATATGGC
KASP8	438072F2	TAGTAAAAATGGGGTTTTATATGGG
	438072R	TCGATACAAATCGATACTTTGGTCCAATC

	295147F1	AAGATGTTTGGAACCATTGCTCTAA
KASP9	295147F2	AAGATGTTTGGAACCATTGCTCTAG
	295147R	TACATATGATTATCTTGCAGTGATGCTGC
	364053F1	TACCACTGTCACTGCTTCTGCAGCT
KASP10	364053F2	TACCACTGTCACTGCTTCTGCAGCA
	364053R	CCCTTGAGCACTGCCAGGGCGCCCACGCC
	364054F1	ATTTCAGACAGCATTTCAATTTTCA
KASP11	364054F2	ATTTCAGACAGCATTTCAATTTTCC
	364054R	CTGTGGGAAGCTTTTTGCCCACTCTTGAG
	21376F1	AACTGGAGTCCTGTCTCGTACCTTG
KASP12	21376F2	AACTGGAGTCCTGTCTCGTACCTTA
	21376R	ATATTTATTTAATTAAGATATAATCGTCG
	21390F1	TGTATGTAGATGATATAAGCGGCAG
KASP13	21390F2	TGTATGTAGATGATATAAGCGGCAA
	21390R	AGAAGTTAGAAGATTTTTGCTTTTATTAG
	128280F1	AGTCAATATTGCCAAGATTAGCTTC
KASP14	128280F2	AGTCAATATTGCCAAGATTAGCTTA
	128280R	CCAATATATACTTTATAGGGGAGGTCATG
	254802F1	GTTTAGGTGCTTCAGAAAATTCCTC
KASP15	254802F2	GTTTAGGTGCTTCAGAAAATTCCTA
	254802R	CATTTTCCCTTATTTATCTCTTTCTATAC
	121779F1	ACCACAAGAAGGCTGAAAGATTAAA
KASP16	121779F2	ACCACAAGAAGGCTGAAAGATTAG
	121779R	TCTCTTTGTATTTTATATATACTTTGTAT
KASP17	38868F1	GTCAGCGCGCGACACGCAATGTCCC
	38868F2	GTCAGCGCGCGACACGCAATGTCCT

	38868R	GACTCTCAGACGCGCTCGGGCATCAGTGC
	791821F1	TATAAGTACTGGTCTCGTCACTAAA
KASP18	791821F2	TATAAGTACTGGTCTCGTCACTAAT
	791821R	GGACGTCATGCGCGAGATGGACGAATTTG
	635323F1	TCATCAGAAAACACGCCACGGTCTC
KASP19	635323F2	TCATCAGAAAACACGCCACGGTCTT
	635323R	CCGCCCTTTCTCTAGGAGGTATGAGGATC
	635082F1	ATAGGAGCCTCCAATGTCATACCAC
KASP20	635082F2	ATAGGAGCCTCCAATGTCATACCAT
	635082R	AAAGCTCCTCAGTATACCCTTTTACCCAT
	635340F1	AGGAAATTCACTAGGCATTGTTTCT
KASP21	635340F2	AGGAAATTCACTAGGCATTGTTTCA
	635340R	CTGAAATCAAAATCAATTTGCAGAAGGGA
	635341F1	CAATTTGCAGAAGGGAAGTGATACC
KASP22	635341F2	CAATTTGCAGAAGGGAAGTGATACT
	635341R	TGATGTTTTCTGTAGGAAATTCACTAGGC
	635106F1	AGGATGATGATAATGATGAGAATCG
KASP23	635106F2	AGGATGATGATAATGATGAGAATCA
	635106R	TACTGTTGTTATTATTGTTATTATCATTA
	162844F1	ACTTTCATGCTTGTGTGACTTTTAC
KASP24	162844F2	ACTTTCATGCTTGTGTGACTTTTAT
	162844R	ATTCGTGGTTATATAATAAGGCGATCATA
	162876F1	TCGGAATGATAGTGATAATGGTATT
KASP25	162876F2	TCGGAATGATAGTGATAATGGTATC
	162876R	TACTGTTGTTATTATTGTTATTATCATTA
	453541F1	GTTTAGGTGCTTCAGAAAATTCCTG
KASP26	453541F2	GTTTAGGTGCTTCAGAAAATTCCTA
	453541R	CATTTTCCCTTATTTATCTCTTTCTATAC

LvHEATR1	q *LvHEATR1*-F1	TTAGTTGTCGCCGAGTCAGTGG
	q *LvHEATR1*-R1	GCTTGTGCGTGGCTGTGAGA
	q *LvHEATR1*-F2	TGCTGGAGGTTGTGACATCTGA
	q *LvHEATR1*-R2	GCTGCTGGCAAGTGGTAGAAGA
β-actin	β-actin-F	CCGGCCGCGACCTCACAGACT
	β-actin-R	CCTCGGGGCAGCGGAACCTC
